# Supplementary material for: L-type voltage-gated calcium channel regulation of in vitro human cortical neuronal networks
Source: Sci Rep. 2019 Sep 25;9:13810. doi: 10.1038/s41598-019-50226-9 (PMC6761148; doi:10.1038/s41598-019-50226-9)
Supplement: Supplementary file 1 — Supplementary Information [file 41598_2019_50226_MOESM1_ESM.docx]

# L-type voltage-gated calcium channel regulation of *in vitro* human cortical neuronal networks

**William Plumbly^1^, Nick Brandon^2^, Tarek Z. Deeb^3^, Jeremy Hall^1^ and Adrian J. Harwood^1^***

*^1^ Neuroscience and Mental Health Research Institute, Cardiff University, Cardiff, CF24 4HQ, UK*

*^2^ Neuroscience, IMED Biotech Unit, AstraZeneca, 35 Gatehouse Dr, Waltham, MA 02451, USA*

*^3^ Department of Neuroscience, Tufts University School of Medicine, Boston, MA, USA*

******* *Correspondence: harwoodaj@cardiff.ac.uk*

**Supplementary Information**

**Descriptions of analysis parameters**

**Average spike rate.** Firstly, the average spike rate for each electrode (E) was calculated as

${AvgSpkRate}_{E}= \frac{{Number of spikes}_{E}}{t_{max}}$, where $t_{max}$ is the total length of the recording in seconds. To give a spike rate for the each array *(A),* $Avg{SpkRate}_{A}=median ({AvgSpkRate}_{E1},\ldots,Avg{SpkRate}_{En})$.

**Number of bursts (Single unit).** Single unit bursts were detected using an inter spike interval (IsI) approach. In all cases, the minimum number of spikes required for a burst was set at 3. Determining the threshold for the maximum IsI interval was achieved by plotting histograms of the total IsIs for each array analysed. Thresholds were determined as the nearest 50 ms greater than the major short interval peak + 50 ms. This threshold was fixed for every set of experiments, not every array and as such was a compromise based on the firing properties of the cohort of arrays being analysed. The maximum IsI interval used throughout was 300 ms.

**Synchronised bursts**

Synchronised busting parameters were based upon the creation of array wide spike detection rates (ADSR). ASDRs were calculated by firstly dividing individual electrode data into 200 ms bins and counting the number of detected spikes within that bin. The bin ASDR was then determined by summing the number of spikes seen in each 200 ms bin culture-wide. ASDR *plots* were then be created by plotting the total number of spikes in each bin serially for the duration of the recording.

Individual synchronised bursts (SBs) were detected from the ASDR data. SB thresholds were first determined over which peaks had to appear: $SB Thr= \frac{MaxASDR}{100}\times n$, where max ASDR is the maximum ASDR for the recording and $n$ determines the threshold level. The threshold level was set at 30% for all experiments. Individual SBs were detected using the findpeaks function of Matlab on the ASDR data, where peaks had to appear over the SB threshold. Parameters for this function were kept constant throughout the experiments. From this function the start and end of each SB was determined, which allowed the calculation of **SB length** and **SB interval** for each detected SB in a recording. As before, the average SB interval / SB length for each array was determined as the median value from all SBs.

The **SB firing rate** was calculated by first determining the rate of firing for each SB in a recording: ${SB firing rate}_{SBn}= \frac{Number of spikes in SBn}{{SB length}_{SBn} (in seconds)}$. The SB firing rate for the array recording was calculated as: $median ({SB firing rate}_{SB1},\ldots,{SB firing rate}_{SBn})$.


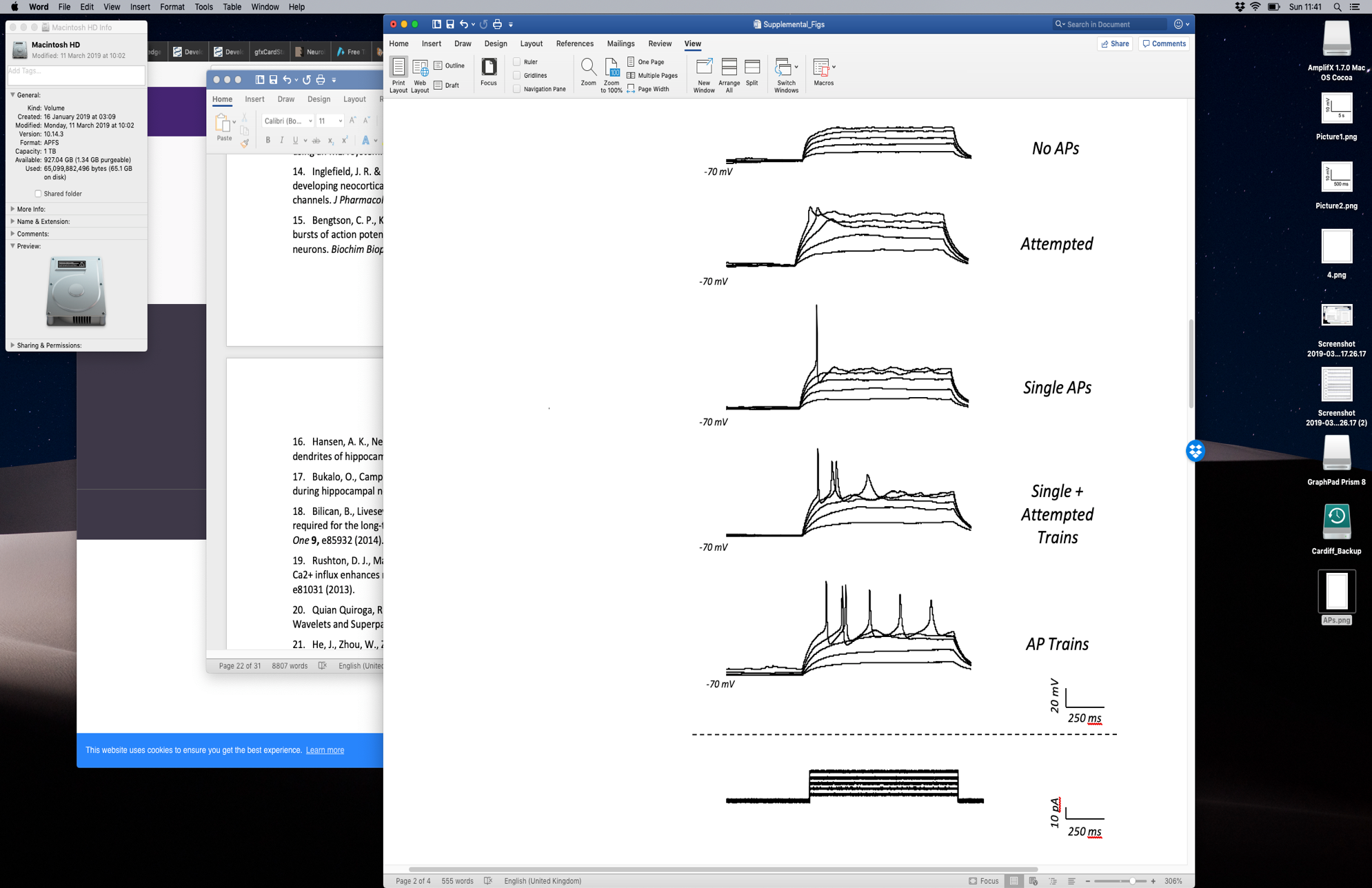

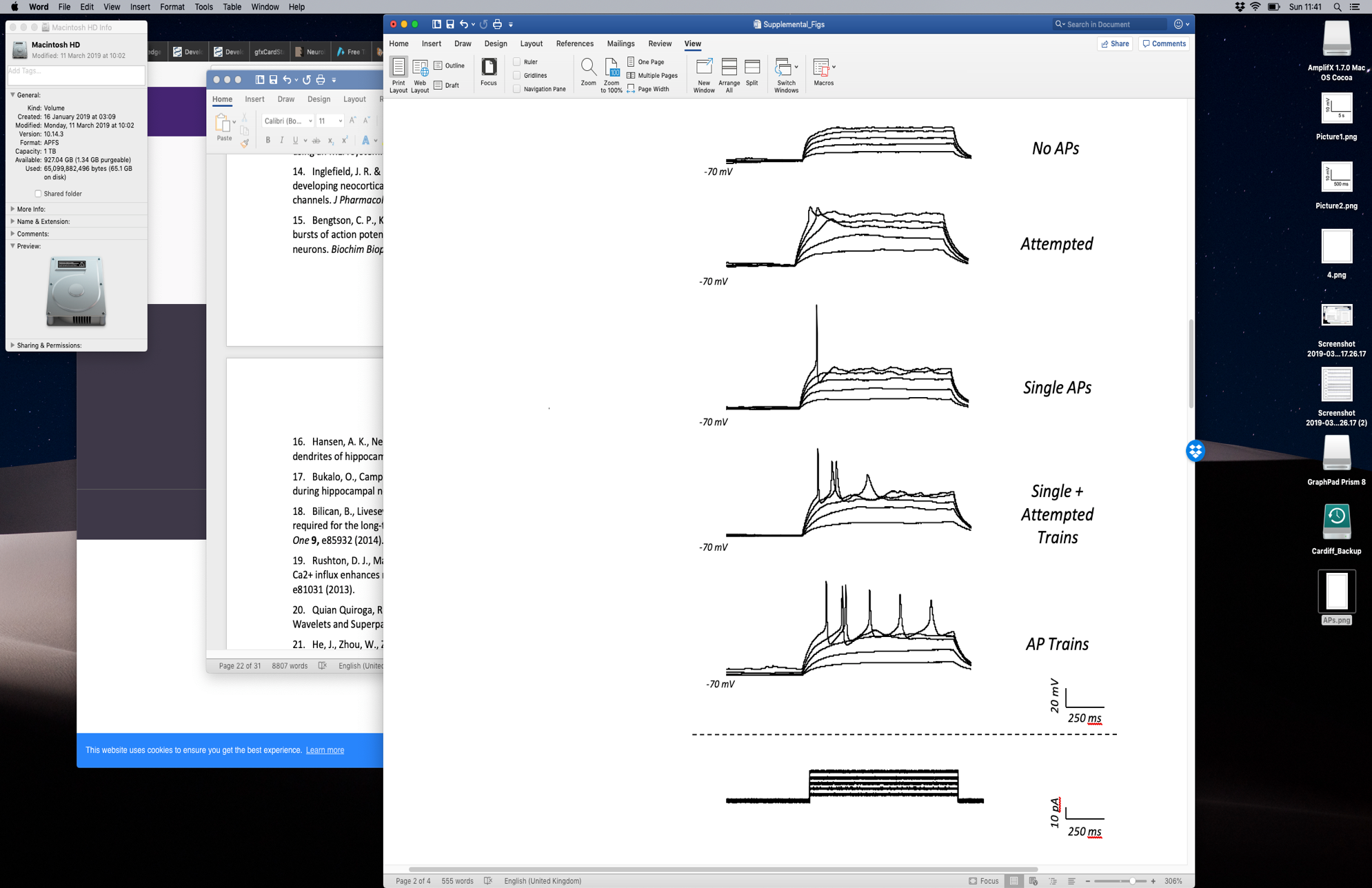


**Figure S1. Examples of different characteristic traces of single neurons in response to current injections during patch-clamp experiments.** The iPSC derived cortical neurons used in this study were coded into categories based upon their response to increasing current injections. Cells were categorised based upon the appearance of no action potentials; attempted action potentials where neurons produce excitable events below 0 mV; Single action potentials with peaks > 0 mV; and action potential trains with at least 2 full action potentials as defined above.

**Figure S2. Early pharmacological profiling of hiPSC derived neuronal cultures.** (A) Raster plots of the same MEA-culture showing activity during acute exposure to (a) inhibitors of AMPA (50 μM CNQX) and NDMA (50 μM APV) receptors and (b) modulators of GABAergic signalling (10 μM bicuculline and 10 μM GABA) at 20 DPP.

**Figure S2. cont’d.** (B) Summary plots showing the average spike rate of cultures of (a) excitatory and (b) inhibitory profiling. Summary plots show means ± SD. * in (b) = p < 0.05 following paired t-test; Wash1 vs GABA, t = 6.425.

**Figure S3. Synchronised bursting in hiPSC derived neuronal cultures is regulated by inhibiting GABA_A_ signalling.** Previous experiments revealed that the rate of firing within and the interval between synchronised burst periods, which develop in neuronal cultures, could be attenuated with acute exposure of the cultures to the competitive GABA_A_ antagonist, bicuculline (pic). To confirm that these observations were indeed the result of GABAergic inhibition, cultures were exposed to the non-competitive GABA_A_ inhibitor picrotoxin (Pic; 5 μM). Acute exposure to picrotoxin caused a notable but insignificant reduction in the basal firing rate, a significant increase in the number of SBs and a significant decrease in the interval between these bursts. (A) raster and ASDR plots showing the response of the same MEA culture before, during and after picrotoxin exposure. (B) Summary plots showing the basal firing rate (a), number of synchronised bursts (b) and the interval between the bursts (c). All plots in B show means ± SD. * in B (b&c) = p<0.05 following paired t-tests (Before vs pic).
